# ROS Induce β-Carotene Biosynthesis Caused by Changes of Photosynthesis Efficiency and Energy Metabolism in *Dunaliella salina* Under Stress Conditions

**DOI:** 10.3389/fbioe.2020.613768

**Published:** 2021-01-15

**Authors:** Yimei Xi, Fantao Kong, Zhanyou Chi

**Affiliations:** School of Bioengineering, Dalian University of Technology, Dalian, China

**Keywords:** *Dunaliella salina*, ROS, β-carotene, carotenogenesis, transcriptomic analysis

## Abstract

The unicellular alga *Dunaliella salina* is regarded as a promising cell factory for the commercial production of β-carotene due to its high yield of carotenoids. However, the underlying mechanism of β-carotene accumulation is still unclear. In this study, the regulatory mechanism of β-carotene accumulation in *D. salina* under stress conditions was investigated. Our results indicated that there is a significant positive correlation between the cellular ROS level and β-carotene content, and the maximum quantum efficiency (*F*_*v*_*/F*_*m*_) of PSII is negatively correlated with β-carotene content under stress conditions. The increase of ROS was found to be coupled with the inhibition of *F*_*v*_*/F*_*m*_ of PSII in *D. salina* under stress conditions. Furthermore, transcriptomic analysis of the cells cultivated with H_2_O_2_ supplementation showed that the major differentially expressed genes involved in β-carotene metabolism were upregulated, whereas the genes involved in photosynthesis were downregulated. These results indicated that ROS induce β-carotene accumulation in *D. salina* through fine-tuning genes which were involved in photosynthesis and β-carotene biosynthesis. Our study provided a better understanding of the regulatory mechanism involved in β-carotene accumulation in *D. salina*, which might be useful for overaccumulation of carotenoids and other valuable compounds in other microalgae.

## Introduction

Carotenoids are light-harvesting pigments that act as antioxidant molecules. Among the carotenoids, β-carotene is a high-value carotenoid that can be produced in many marine animals, higher plants, and microorganisms including microalgae. Due to its strong pigmentation function, powerful antioxidative activity, and broad beneficial effects on human health, β-carotene possesses a wide range of applications in feed, food, and nutraceutical and pharmaceutical industries, which has attracted great attention (Jin and Melis, 2003; Combe et al., 2015; Liang et al., 2019). However, humans cannot synthesize β-carotene and must obtain it through diet. In 2015, the global β-carotene market was approximately estimated to be US$ 432 million with 36% revenues from microalga-derived natural β-carotene (Nethravathy et al., 2019).

Microalgae are a group of photosynthetic microorganisms and are sources of numerous value-added products, such as lipids, carbohydrates, and carotenoids (Chew et al., 2017). The green microalga *Dunaliella salina* (thereafter *D. salina*) can synthesize an extremely large amount of β-carotene (up to 10% of the dry weight) (Harvey and Xu, 2019). The high carotene contents in *D. salina* make it a promising cell factory for the large-scale production of natural β-carotene. Nevertheless, *D. salina* has inherent limitations such as slow growth rate and low biomass yield. Moreover, the regulatory mechanism of β-carotene production in *D. salina* is not clear, which hinders the economically feasible production of β-carotene at industrial level.

It has been reported that an increase in intracellular β-carotene content is often accompanied by a high level of reactive oxygen species (ROS) in microalgal cells which are suffering from abiotic stresses, such as high light, high salinity, and nutrient deprivation (Cowan et al., 1992; Lamers et al., 2008; Ye et al., 2008). ROS mainly include hydrogen peroxide (H_2_O_2_), superoxide (O2^−^), and hydroxyl radical (OH^·^). It was reported that ROS can act as signals to trigger various cellular events, i.e., activation of many metabolic pathways through phosphorylation cascades and the oxidation of key signaling molecules (Shi et al., 2017; Zhang L. et al., 2019). As signaling molecules, the moderate level of ROS is beneficial for the cells to regulate gene expression and initiate self-protective mechanisms in response to environmental changes (Cowan and Rose, 1991; Liu et al., 2012). However, a high level of ROS accumulated during stress will damage DNA, proteins, lipids, and photosynthetic pigments in microalgae (Liu et al., 2012).

ROS are generally generated from chloroplast and mitochondrial electron transport flows due to excitation of O_2_ by excessive electrons in microalgae. The chloroplast is the primary compartment for β-carotene formation (Cowan et al., 1992; Kamalanathan et al., 2017). ROS were produced accompanied with β-carotene formation under stress conditions, which can alleviate the oxidative damage to the photosynthetic system in microalgae (Henriquez et al., 2016). Shaish et al. (1993) reported that the photosynthetically produced ROS are involved in triggering massive β-carotene accumulation in *D. bardawil*. However, the molecular mechanism is still not clear. Under abiotic stress conditions, e.g., high light (HL), nitrogen deprivation (N–) and high salinity (HS), and β-carotene overaccumulation accompanied with high level of ROS were found in *D. salina*, respectively (Liu et al., 2012; Mirshekari et al., 2019; Zhao et al., 2019). However, there is also still a lack of understanding regarding the regulatory mechanism of ROS on β-carotene accumulation in *D. salina* under these stress conditions. Moreover, the relationship between photosynthesis activity and β-carotene accumulation is still poorly understood.

Here we investigated the relationship between ROS content and β-carotene accumulation in *D. salina* under different stress conditions (i.e., high light, high salinity, and nitrogen starvation), and the correlation between photosynthesis activity and β-carotene accumulation was also analyzed using Spearman correlation analysis. Additionally, the differential expression of key genes involved in photosynthesis and β-carotene biosynthesis pathways of the cells cultivated with H_2_O_2_ supplementation was also examined through transcriptomic analysis. In this work, we found that ROS play an important role in β-carotene metabolism through fine-tuning the key genes that are involved in photosynthesis and β-carotene biosynthesis in *D. salina* under stress conditions. Our work might shed light on future research aiming to enhance β-carotene accumulation in *D. salina* and other microalgae.

## Materials and Methods

### Algal Strain and Culture Conditions

*Dunaliella salina* strain (CCAP 19/18) was purchased from the Culture Collection of Algae and Protozoa (Windermere, United Kingdom). The strain was previously maintained in the medium of modified Artificial Sea Water (ASW), which was composed of 1.5 M NaCl, 5 mM KNO_3_, 0.45 mM MgCl_2_·6H_2_O, 0.05 mM MgSO_4_·7H_2_O, 0.3 mM CaCl_2_·2H_2_O, 0.13 mM K_2_HPO_4_, 0.02 mM FeCl_3_, 0.02 mM EDTA, and 1 mL of trace element stock containing 50 mM H_3_BO_3_, 10 mM MnCl_2_·4H_2_O, 0.8 mM ZnSO_4_·7H_2_O, 0.8 mM CuSO_4_·5H_2_O, 2 mM NaMoO_4_·2H_2_O, 1.5 mM NaVO_3_, and 0.2 mM CoCl_2_·6H_2_O, and the pH was adjusted to 7.5 (Doddaiah et al., 2013). This strain was maintained in 500-mL conical flasks at 50 μmol photons·m^−2^·s^−1^ light intensity in this study. To test the effect of H_2_O_2_ supplementation on β-carotene accumulation in cells, different concentrations of H_2_O_2_ (0, 0.5, 1.0, 1.5, and 2.0 mM) were added to algal cultures in ASW medium.

The cells were cultivated in air-lift laboratory-scale flat photobioreactors (0.22 × 0.22 × 0.025 m) filled with 1.0 L of culture medium under the following five different culture conditions: nitrogen starvation (N–, ASW medium without KNO_3_), high light (HL, 2,000 μmol photons·m^−2^·s^−1^), high salinity (HS, 3.0 M NaCl), and optimal growth condition (N+, control). Unless otherwise noted, all the cells were cultured in ASW medium at 25°C aerated with filter-sterilized air at 0.5 vvm and illuminated at 100 μmol photons·m^−2^·s^−1^. The different culture conditions were shown in Table 1.

**Table 1:** The different culture conditions used in this study.

**Culture conditions**	**Abbreviations**	**Light intensity (μmol·m^−2^·s^−1^)**	**NaCl (M)**	**KNO_**3**_ (mM)**
Control	N+	100.0	1.5	5.0
Nitrogen starvation	N–	100.0	1.5	0.0
High salinity	HS	100.0	3.0	5.0
High light	HL	2000.0	1.5	5.0

### Biomass and β-Carotene Determination

Dry weight was determined as previously described (Chi et al., 2016). Briefly, 10.0-mL cultures were filtered using pre-weighed Whatman GF/C filters (47 mm diameter) and washed three times with 2.0 mL 0.5 M ammonium bicarbonate and then were dried at 60°C for 16 h in an incubator. The dried biomass (g·L^−1^) of the microalgal cells was then gravimetrically measured.

### β-Carotene Extraction and Determination

β-Carotene was extracted and measured following the method previously described (Zhu et al., 2018, Kleinegris et al., 2010). Briefly, 1.0 mL of cell culture at exponential phase was harvested at 10,000 g for 2 min and resuspended in 3.0 mL dodecane and 9.0 mL of methanol. After centrifugation for 2 min at 10,000 g, the dodecane-containing lipophilic carotenoids (upper layer) were obtained and measured with a spectrophotometer (Jasco V-530, JASCO Corporation, Japan) at 453 and 665 nm with dodecane as a reference. The carotene concentration was calculated as Equation (1):

(1) $$\begin{array}{r} {\text{C}}_{\text{β}-car}\left(\text{mg}\cdot {\text{L}}^{-1}\right)=\left({\text{A}}_{453}-{\text{A}}_{665}/3.91\right)\times 3.657\times 3\times \text{X} \end{array}$$

where (A_453_-A_665_/3.91) is the absorbance of β-carotene corrected for chlorophyll contamination, 3.657 is the calibration factor derived from HPLC analysis of β-carotene concentration, 3 is the amount of milliliters of dodecane added for extraction, and X is the dilution factor to measure absorbance on spectrophotometer (Kleinegris et al., 2010, 2011).

The amount of β-carotene in the algal biomass was calculated according to Equation (2).

(2) $$\begin{array}{r} \beta -\text{carotene}\left(\%\right)=\frac{C_{\beta -car}\times 10}{DW} \end{array}$$

where C_β−*car*_ is the β-carotene content (mg·L^−1^), and DW is the cell dry weight (g·L^−1^) (Zhu et al., 2018).

### Determination of ROS and H_2_O_2_ Levels

The ROS levels were determined using the ROS assay kit (Beyotime Institute of Biotechnology, China) as previously reported (Kong et al., 2018). Briefly, 10 μM of 2′,7′-dichlorofluorescein diacetate (DCFH-DA) was added to 1.0 mL samples containing 1.0 × 10^6^ cells and incubated for 20 min in the dark. The samples were washed twice and resuspended in 1.0 mL fresh medium. The fluorescence emission spectra were read at 525 nm (with excitation at 500 nm) using a fluorescence spectrophotometer (F-4500, Hitachi, Japan) (Liu et al., 2012).

The H_2_O_2_ content was measured using a commercial H_2_O_2_ assay kit (NJJCBIO, China) as previously described (Zhang L. et al., 2019). Briefly, 1.0 × 10^6^ fresh algal cells were homogenized in an ice bath and centrifuged at 10,000 g for 3 min, and H_2_O_2_ concentration was measured following the manufacturer's protocols.

### Chlorophyll Fluorescence Analysis

The chlorophyll fluorescence was measured by a Dual Pulse Amplitude Modulated Fluorometer (Water-PAM Heinz Walz GmbH, Effeltrich, Germany). Dark adaption was carried out for 10 min before applying a saturating pulse (0.6 s, 1,400 μmol·m^−2^·s^−1^) to measure the maximal quantum yield of PS II (*F*_*v*_*/F*_*m*_), non-photochemical quenching (NPQ) and actual quantum yield of PS II (*F/F*_m_^′^) (Chi et al., 2016; Liu et al., 2018).

### RNA-Seq and Differentially Expressed Gene Analysis

Total RNAs were, respectively, extracted from three biological replicates of *D. salina* cells cultivated with or without H_2_O_2_ supplementation using a Total RNA Extraction System (Takara, Japan) following the previous method (Liang et al., 2020). The RNA concentration and integrity were assessed using NanoDrop 2000 (Thermo) and an Agilent 2100 Bioanalyzer (Agilent Technologies, CA, USA). The mRNA was purified and fragmented to about 200 nt. These fragments were used as templates to synthesize cDNA. After purification, their sequencing was performed in Novogene Bioinformatics Technology Co. (Beijing, China).

RNA-seq data was analyzed by Novogene Bioinformatics Technology Co. (Beijing, China). Clean reads obtained from editing raw reads were mapped onto unigene sequences using Bowtie2-2.2.3. The expected number of Fragments Per Kilobase of transcript sequence per Millions base pairs sequenced (FPKM) was performed to quantify gene expression levels, and DESeq R package was used to analyze the differentially expressed genes (DEGs) in *D. salina* cells between control and H_2_O_2_-induced samples with a significantly differential expression at *p* < 0.05 and fold change ≥1. The Kyoto Encyclopedia of Genes and Genomes (KEGG) pathways for DEGs were annotated by KEGG automatic annotation server (Liang et al., 2020).

### Statistical Analysis

All the experiments were carried out in three biological replicates, and the data were presented as the mean value ± standard division (SD). Correlation between ROS and β-carotene content was analyzed using Spearman correlation analysis and the statistical analyses were conducted using SPSS 19.0 (SPSS, Chicago, IL, USA).

## Results and Discussion

### The β-Carotene Production Was Enhanced Under Stress Conditions in *D. salina*

**Figure 1:**
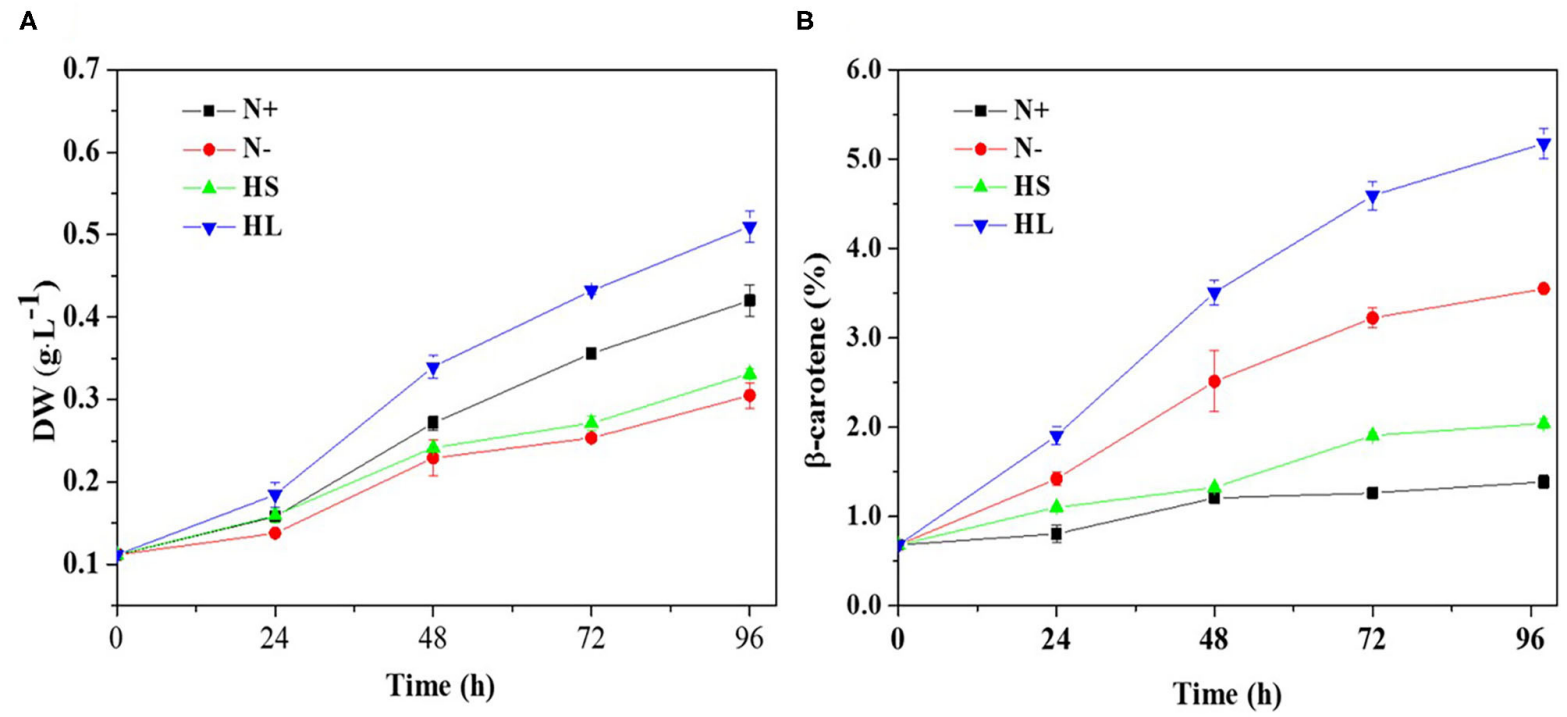
Time profiles of the growth and β-carotene accumulation **(A,B)** of *D. salina* cells cultivated under different stress conditions. DW, dry weight; N+, optimal growth condition (control); N–, nitrogen starvation; HS, high salinity; HL, high light. Plotted data are the averages ± standard division (SD) of three biological replicates.

To date, several studies have reported the effect of different culture conditions (e.g., HL, HS, and N-) on the β-carotene content in *D. salina*, respectively (Liu et al., 2012; Mirshekari et al., 2019; Zhao et al., 2019). However, it is difficult to directly compare the exact results of these studies, mainly due to various initial cell numbers inoculated and different illumination techniques applied. To study whether various stress conditions result in the same oxidative stress for carotenoid accumulation, we compared the effect of different stress conditions on β-carotene and biomass production in *D. salina*. We inoculated the cells with the same initial biomass density (0.11 g·L^−1^) and cultivated them in photobioreactors that enable well-defined light regimes and remain constant cultivation conditions throughout experiments. The lower biomass concentrations were observed under N- and HS stress conditions compared with optimal growth conditions (N+) (Figure 1A). For example, the biomass concentrations were, respectively, 0.31 and 0.33 g·L^−1^ under the N– and HS conditions upon 96 h, which were significantly lower than that of the N+ condition (0.42 g·L^−1^). However, under HL conditions *D. salina* has a higher biomass concentration (e.g., 0.51 g·L^−1^ upon 96 h) compared with the N+ condition (Figure 1A). Although the cell growth was arrested under N– and HS conditions, β-carotene accumulation was enhanced under all the stress conditions tested. The maximal β-carotene content was observed under HL (5.18%), followed by 3.55 and 2.04% under N- and HS conditions, which are significantly higher than that under N+ conditions (1.38%) (Figure 1B). These results suggest that the slower growth rate or the higher light intensity can enhance the production level of β-carotene. The increased β-carotene levels might be due to the response to these stress conditions.

In *D. salina*, β-carotene is largely accumulated under N- conditions (Lamers et al., 2012). β-Carotene accumulation was also enhanced under HL or HS conditions (Lamers et al., 2008, 2010; Einali and Valizadeh, 2015). Indeed, we have also found that the production level of β-carotene is higher in those stress conditions tested (Figure 1). Moreover, our results suggested that the content of β-carotene is higher but the biomass is lower under N- and HS stress conditions (Figure 1A). This could be partially due to large amounts of carbon and ATP being channeled from synthesis of energy compounds for growth to the synthesis of metabolites (e.g., carotenoids) (Liu et al., 2018). Noticeably, the biomass and β-carotene accumulation are both higher in HL conditions compared with other cultivation conditions tested (Figure 1). This could be due to the fact that more energy was transferred to biomass production and β-carotene synthesis under HL conditions in *D. salina* (Wu et al., 2016; Habiby et al., 2018; Song et al., 2018). It is likely that HL is a more potent inducer of β-carotene overproduction than nitrogen depletion (Figure 1B). However, in terms of energy cost, β-carotene production through nitrogen depletion still appears more promising, since 20 times less light energy was applied than that in the HL condition (Table 1). In contrast to the other stress conditions tested, the intracellular β-carotene content was only increased to 2.04% upon 96 h after high-salinity treatment (Figure 1B). This result indicated that salinity-induced β-carotene accumulation was very limited in *D. salina*. Recently, it was also reported that high-salinity stress does not significantly promote the accumulation of β-carotene or even has adverse effects in an isolated *D. salina* strain (GY-H13) (Zhu et al., 2020), which is similar with reports in other microalgae, such as *Dunaliella parva* (Shang et al., 2018). Taken together, our results indicated that β-carotene production was enhanced under all the stress conditions tested, even though the increased levels of β-carotene and growth rate in response to stimuli derived from different stress are varied.

### Correlation Between Photosynthesis Activity and β-Carotene Accumulation

**Figure 2:**
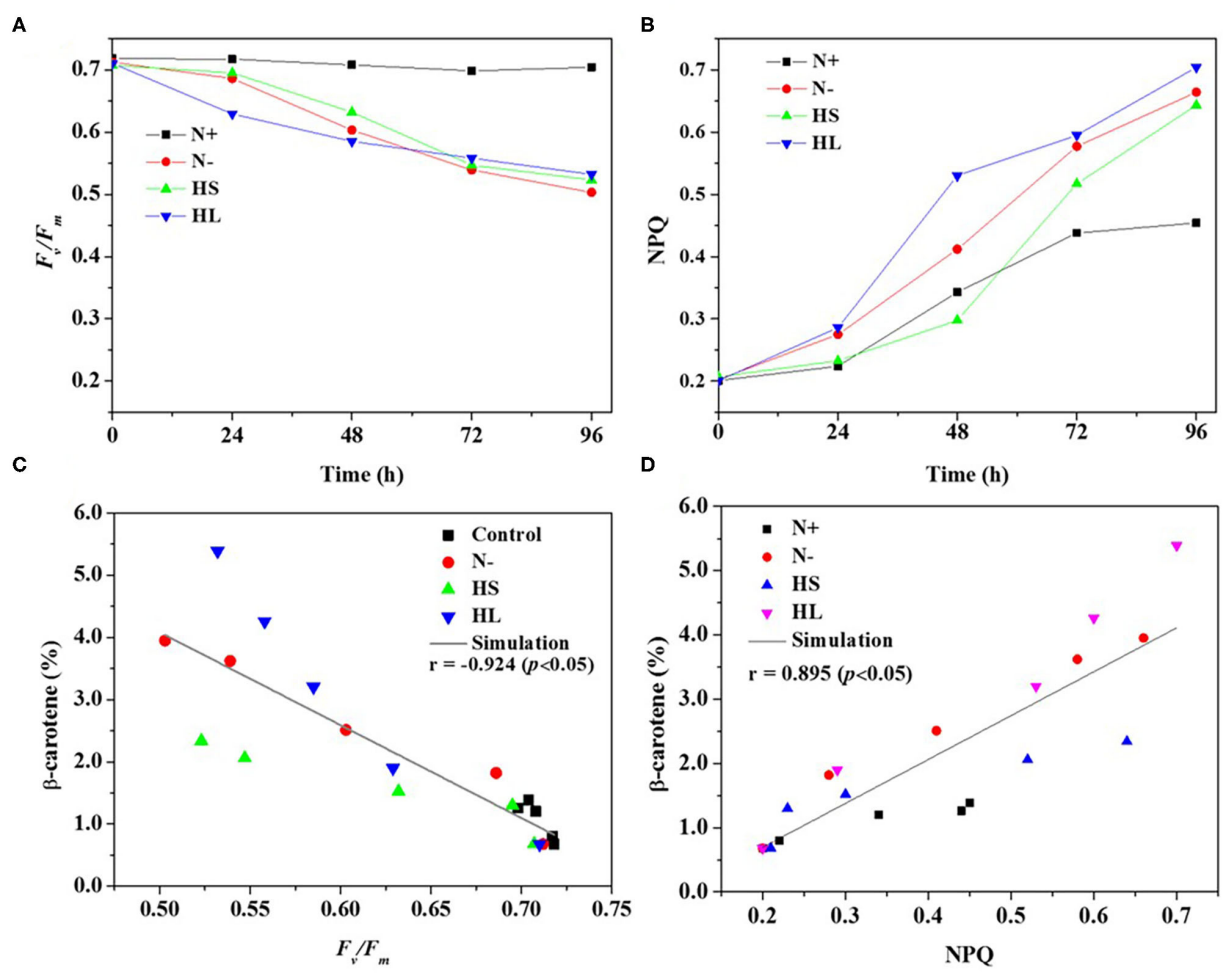
The profiles of the *F*_*v*_*/F*_*m*_ and NPQ **(A,B)** evaluated over time, and the correlation of the *F*_*v*_*/F*_*m*_ and NPQ with corresponding carotene content **(C,D)** under different culture conditions, respectively. *F*_*v*_*/F*_*m*_, maximum quantum efficiency of PS II; NPQ, non-photochemical quenching; N+, optimal growth condition (control); N-, nitrogen starvation; HS, high salinity; HL, high light. Plotted data are the averages ± SD of three biological replicates.

Carotenoids are essential components of photosynthetic organisms including algae. We examined the relationship between photosynthesis activity and β-carotene accumulation. Although under stress conditions (i.e., HL, N–, and HS) the β-carotene content was increased (Figure 1B), the maximal quantum yield of PS II (*F*_*v*_*/F*_*m*_) was decreased significantly and the non-photochemical quenching (NPQ) was enhanced (Figures 2A,B).

Furthermore, Spearman correlation analysis was used to investigate the correlation between photosynthesis activity and β-carotene accumulation under stress conditions. The maximal Spearman correlation coefficient (−0.924, *p* < 0.05) was observed by plotting the *F*_*v*_*/F*_*m*_ against the corresponding β-carotene content (%) (Figure 2C), while the coefficient of 0.895 (*p* < 0.05) was obtained when plotting NPQ (Figure 2D). These results show that *F*_*v*_*/F*_*m*_ were negatively while NPQ were positively correlated with β-carotene accumulation under different stress conditions. It is found that *F*_*v*_*/F*_*m*_ can be regarded as a stress indicator (Cao et al., 2019); the decrease of *F*_*v*_*/F*_*m*_ suggested that the photosynthesis activity was arrested under N starvation, HL, and HS stress conditions. NPQ is a protection process that thermally dissipates excessive light energy absorbed by photosynthesis pigment (Wang et al., 2016). It was reported that ROS produced under stress conditions can decrease photosynthetic activity through the peroxidation of lipids in the thylakoids, damage of PSII complex, and decrease in overall activity of the electron transport chain of PSII (Salguero et al., 2003; Zhang L. et al., 2019). Therefore, ROS generation may be one of the major factors accounting for the decrease of photosynthesis efficiency (the effective photochemical efficiency of PSII reaction centers) in *D. salina* under stresses conditions.

### Correlation Between ROS Production and β-Carotene Accumulation

**Figure 3:**
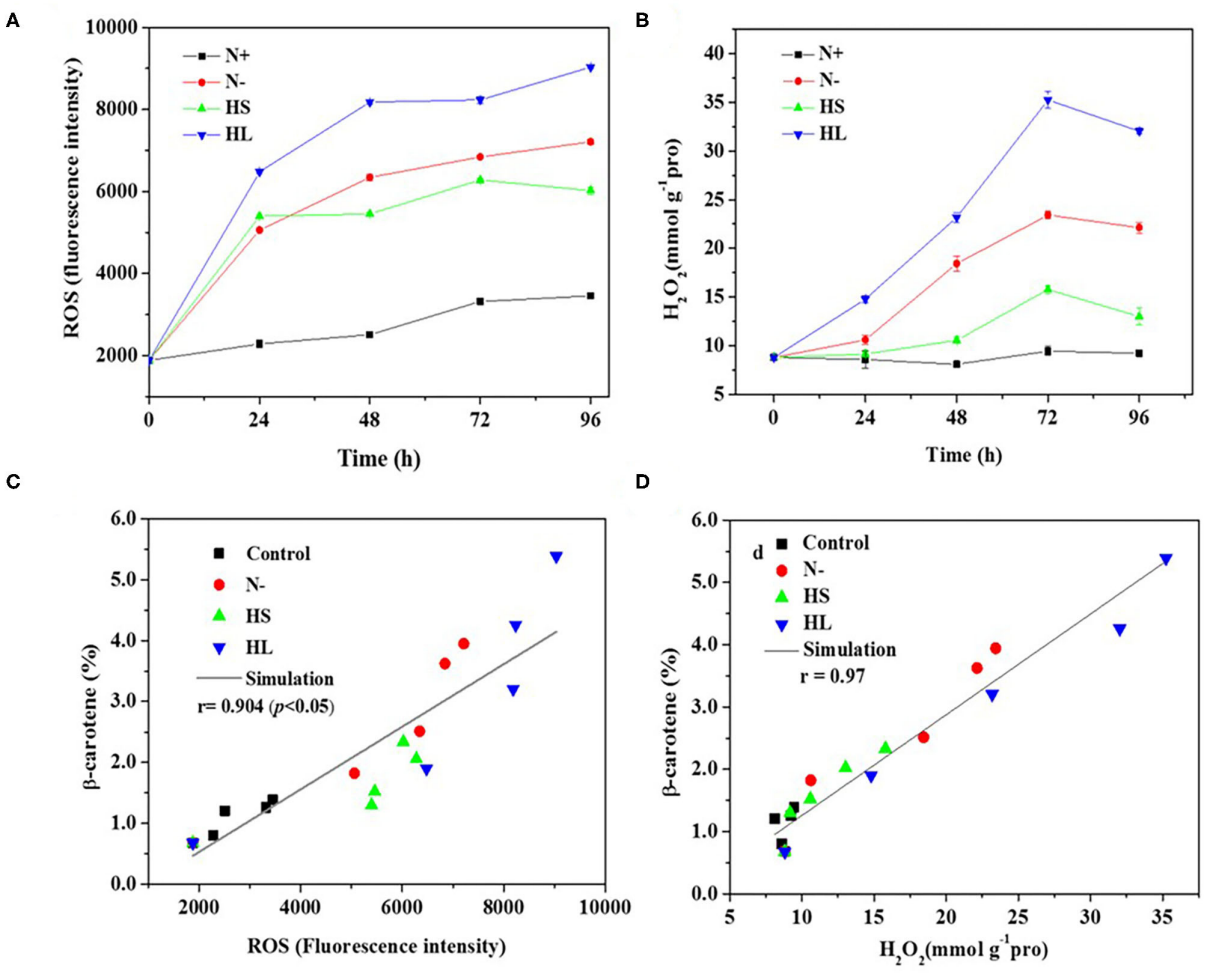
The intracellular contents of ROS and H_2_O_2_ **(A,B)**, and relationship of total ROS and H_2_O_2_ with corresponding β-carotene content **(C,D)** under different stress conditions, respectively. N+, optimal growth condition (control); N–, nitrogen starvation; HS, high salinity; HL, high light. The experiments were carried out in triplicate and the data were presented as the mean value ± SD.

In the photosynthetic microorganisms, the imbalance between light harvesting and energy utilization during stress conditions will result in the production of ROS, due to overexcitation of chlorophyll molecules (Shi et al., 2017). Then, the contents of total ROS and representative ROS (e.g., H_2_O_2_) were investigated under the aforementioned stress conditions. We found that the contents of total ROS and H_2_O_2_ are significantly higher under all the stress conditions compared with normal condition (N+) (Figures 3A,B). However, the ROS and H_2_O_2_ levels are varied among all stress conditions tested with the highest level under the HL condition (Figures 3A,B). The plateau period of ROS and H_2_O_2_ contents appeared at 48 and 72 h, respectively (Figures 3A,B), which indicate that other ROS (e.g., O2^·^- and OH^·^) are also promptly produced in the cells under stress conditions.

Although a simultaneous increase in ROS and β-carotene accumulation under stress conditions was observed in *D. salina*, the relationship between ROS production and β-carotene accumulation is currently still unclear. In this study, Spearman correlation analysis was used to investigate the correlation between the two events. Here, four stress conditions were evaluated together rather than examining a single condition to avoid false-positive correlations. The maximal Spearman correlation coefficient (0.90, *p* < 0.01) was observed by plotting the ROS concentration against the corresponding β-carotene content (%) (Figure 3C), while the coefficient of 0.97 (*p* < 0.01) was obtained when plotting H_2_O_2_ (Figure 3D). Generally, if the Spearman correlation coefficient is more than 0.8, it is assumed that there is a strong correlation between two events (Zhang L. et al., 2019). Thus, our results show that intracellular ROS and H_2_O_2_ levels were positively correlated with β-carotene accumulation under different stress conditions. The simultaneous increases of ROS and β-carotene contents under stress conditions were also found in other green microalgae such as *C. reinhardtii* (Sun et al., 2018). Moreover, it was reported that the upregulation of *de novo* carotenoid synthesis in *C. reinhardtii* cells under HL conditions is mediated through H_2_O_2_ (Chang et al., 2013).

Overall, our data indicated that the stress conditions are likely to cause the decrease of *F*_*v*_*/F*_*m*_ accompanied with the high level of ROS generated. Subsequently, the ROS trigger the overaccumulation of β-carotene. The elevated β-carotene content under stress conditions might be due to the stimuli caused by ROS in *D. salina* under stress conditions.

### H_2_O_2_ Supplementation Results in Lower Biomass and β-Carotene Overaccumulation

**Figure 4:**
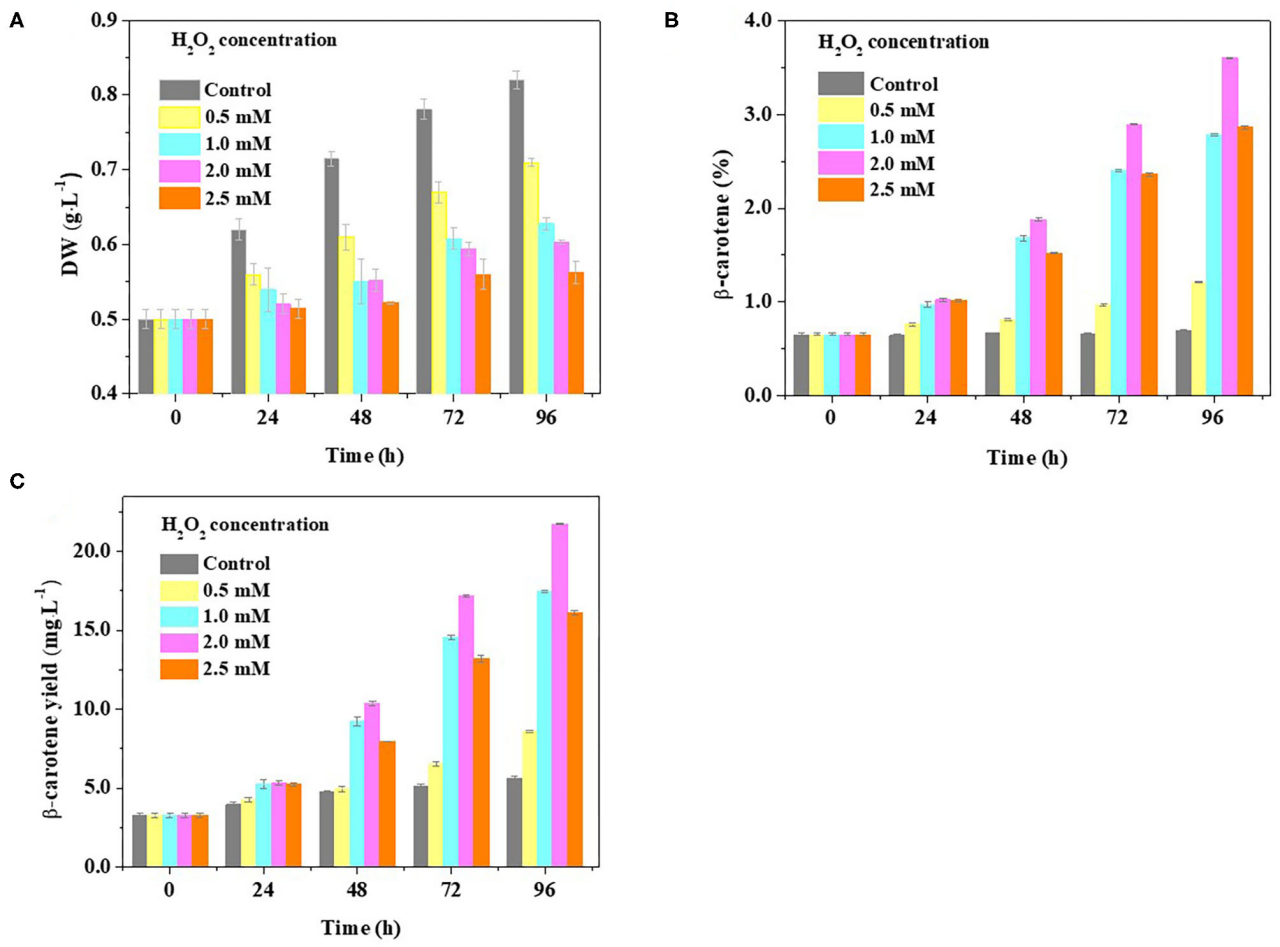
Effect of H_2_O_2_ supplementation on biomass and β-carotene content in *D. salina*. Time-course analyses of the biomass **(A)** and β-carotene content and yield **(B,C)** when the cells were cultivated with H_2_O_2_ supplementation of different concentrations. DW, dry weight. Plotted data are the averages ± SD of three biological replicates.

To further evaluate the idea that β-carotene overaccumulation observed in *D. salina* may result from an increased ROS, we determined the β-carotene contents in the cells cultivated with H_2_O_2_ supplementation. Considering the dual role of H_2_O_2_ on cell physiology and metabolism, the H_2_O_2_ concentration used was optimized in this work based on the ratio of H_2_O_2_ provided by previous reports (Yilancioglu et al., 2014; Zhang et al., 2016). We found that the biomass was reduced while the β-carotene content was increased when the amount of H_2_O_2_ concentration supplemented is increasing (Figures 4A,B). A significant increase in cellular β-carotene concentration (*p* < 0.05) was observed at 2.0 mM H_2_O_2_ upon 96 h (Figure 4B). The β-carotene content was increased to 6.7-fold, and the yield of β-carotene was 3.9-fold higher at 2.0 mM H_2_O_2_ than that under optimal growth conditions (Figures 4B,C). These results suggest that β-carotene accumulation can be enhanced by moderate H_2_O_2_ supplementation.

### Transcriptomic Analysis Reveals Key Genes Involved in β-Carotene Overaccumulation Induced by ROS

**Figure 5:**
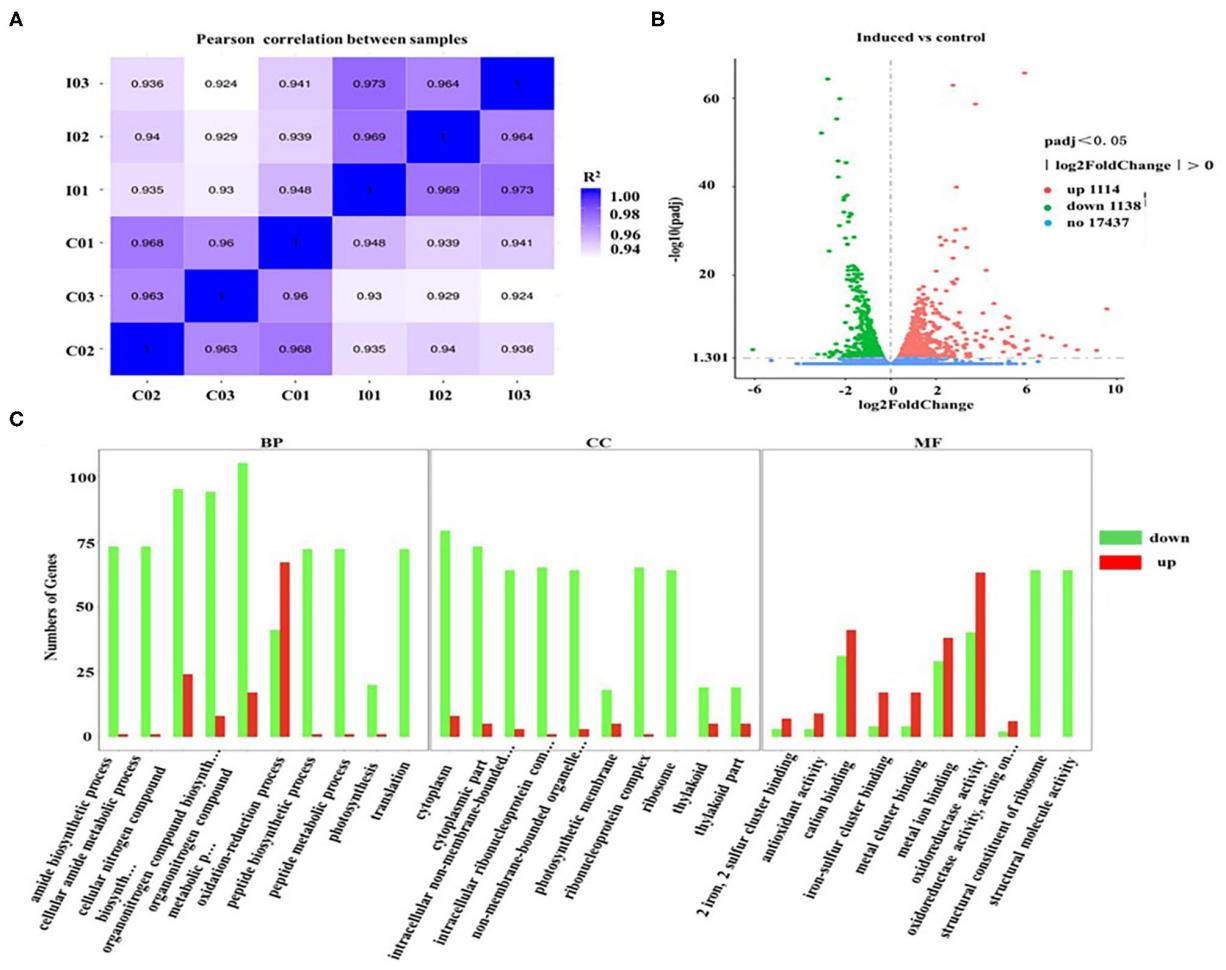
Gene ontology classification analysis of unigenes that are involved in the overaccumulation of β-carotene induced by ROS. **(A)** The correlation plot between different samples. I01, I02, and I03 (2.0 mM H_2_O_2_); C01, C02, and C03 (Control, 0 mM H_2_O_2_). **(B)** MA plot of differentially expressed genes (DEGs). Significantly upregulated and downregulated genes are shown as red and green dots, respectively. Genes with no significant changes are shown as blue spots. **(C)** Gene ontology (GO) classification analysis of DEGs. The X-axis shows the GO function. The Y-axis shows the number of genes detected. Significantly upregulated and downregulated genes are shown as red and green rectangles. BP, biological processes; CC, cellular components; and MF, molecular functions.

To explore the transcriptional response induced by H_2_O_2_, the differentially expressed genes (DEGs) from *D. salina* grown with H_2_O_2_ supplementation (2.0 mM) were investigated by transcriptomic analysis. A high correlation coefficient was obtained within the same treatment condition, while a low correlation coefficient was observed between the different treatment conditions (Figure 5A). These results indicate that the transcriptome data were reliable. There are 2,252 genes with a change of at least 2-fold that we assumed as DEGs, of which 1,114 genes were upregulated, and 1,138 genes were downregulated (Figure 5B).

Furthermore, the gene ontology (GO) classification was performed according to the gene annotations. Most of DEGs were distributed in three GO terms, among which 526, 197, and 679 of DEGs were assigned in the biological process category, cellular component category, and molecular function category, respectively (Figure 5C). In the biological processes, the most active subcategories were the clusters of the “metabolic process” and “cellular process.” The main subcategories in molecular functions were the clusters of “binding” and “catalytic activity.” In the cellular components, most of the detected DEGs were dominantly located in “cell” and “cell part.” Moreover, the genes involved in the biological processes of “reproduction” and “reproductive processes” were significantly downregulated in *D. salina* (Figure 5C), which partially account for the reasons of the increased growth of the cells as shown in Figure 1A.

We found that the β-carotene and ROS contents were increased but photosynthesis activity was reduced during cultivation of *D. salina* (Figures 1, 2) under stress conditions, and therefore, the genes involved in the photosynthesis and β-carotene biosynthesis were analyzed upon H_2_O_2_ supplementation. Most of DEGs involved in photosynthesis pathways (e.g., antenna proteins) were significantly downregulated when H_2_O_2_ was supplemented (Supplementary Figures 1, 2). This result is consistent with a previous study that the major DEGs involved in photosynthesis pathways (e.g., PSII) exhibited a dramatic downregulation with ROS burst in *Dunaliella bardawil* under heat stress conditions (Liang et al., 2020). In other microalgae such as *Chromochloris zofingiensis*, it was also reported that most genes encoding components of PS I, PS II, light-harvesting complexes (LHCs), and Cyt *b*_6_/*f* exhibited dramatic downregulation in response to nitrogen deprivation (Zhang Y. et al., 2019). Furthermore, our results showed that the key genes involved in biosynthesis of carotenoids were upregulated upon H_2_O_2_ supplementation (Supplementary Figure 3). For example, phytoene synthase (PSY), phytoene desaturase (PDS), and zeta-carotene desaturase (ZDS), known as the rate-limiting enzymes during carotenoid biosynthesis (Shang et al., 2018; Zhu et al., 2020), were highly overexpressed upon H_2_O_2_ supplementation (Supplementary Figure 3). These results are consistent with the previous studies that the expressions of *PSY, PDS*, and *ZDS* were upregulated by quantitative RT-PCR validation in *D. salina* under stress conditions (e.g., high light, nutrient deprivation, high salinity), respectively (Coesel et al., 2008; Lv et al., 2016; Zhu et al., 2020). Similarly, in the H_2_O_2_-treated *C. reinhardtii* cells, the transcripts of both *PSY* and *PDS* were also dramatically increased (Chang et al., 2013). The increased expression of these key genes indicated that they might play an important role in the biosynthesis of β-carotene.

The genes involved in starch, sucrose, fatty acids, and amino acid metabolism were also examined. There are 9 genes that are involved in the synthesis of starch and sucrose which were downregulated in *D. salina* cells when cultivated with H_2_O_2_ supplementation (Supplementary Table 1). The expression of 28 genes which are involved in the biosynthesis of amino acids pathway was negatively correlated with H_2_O_2_ (Supplementary Table 2). A similar phenomenon was also found in the previous study that the DEGs responsible for the biosynthesis of amino acids was downregulated in *D. bardawil* under heat stress (Liang et al., 2020). Our transcriptomic data showed that genes such as *FabF, FabD, FabG, FabZ*, and *FabI* that are involved in the fatty acid biosynthesis pathway were downregulated (Supplementary Table 3), while the genes such as *FDH1, ATO1, ACAT*, and *ACSL* that are involved in the fatty acid degradation were highly expressed when H_2_O_2_ was supplemented (Supplementary Table 4). Most of the DEGs involved in fatty acid biosynthesis were also downregulated in *D. bardawil* under heat stress (Liang et al., 2020) and in *C. zofingiensis* upon nitrogen deprivation condition (Zhang Y. et al., 2019; Zhang et al., 2020). Overall, our results indicated that the increased expressions of genes involved in fatty acid degradation and the decreased expression of genes involved in the pathways of fatty acid, amino acid, and starch biosynthesis might facilitate β-carotene overaccumulation in *D. salina* after H_2_O_2_ was supplemented. Moreover, the transcriptome data show that the expressions of key genes involved in the synthesis of energy storage compounds (e.g., starch and lipids) and the photosynthesis activity were decreased, respectively, in *D. salina* cells cultivated with H_2_O_2_ supplementation, which might account for the changes of cellular photosynthetic efficiency and energy metabolism (Liska et al., 2004; Li et al., 2010; Zhao et al., 2019).

**Figure 6:**
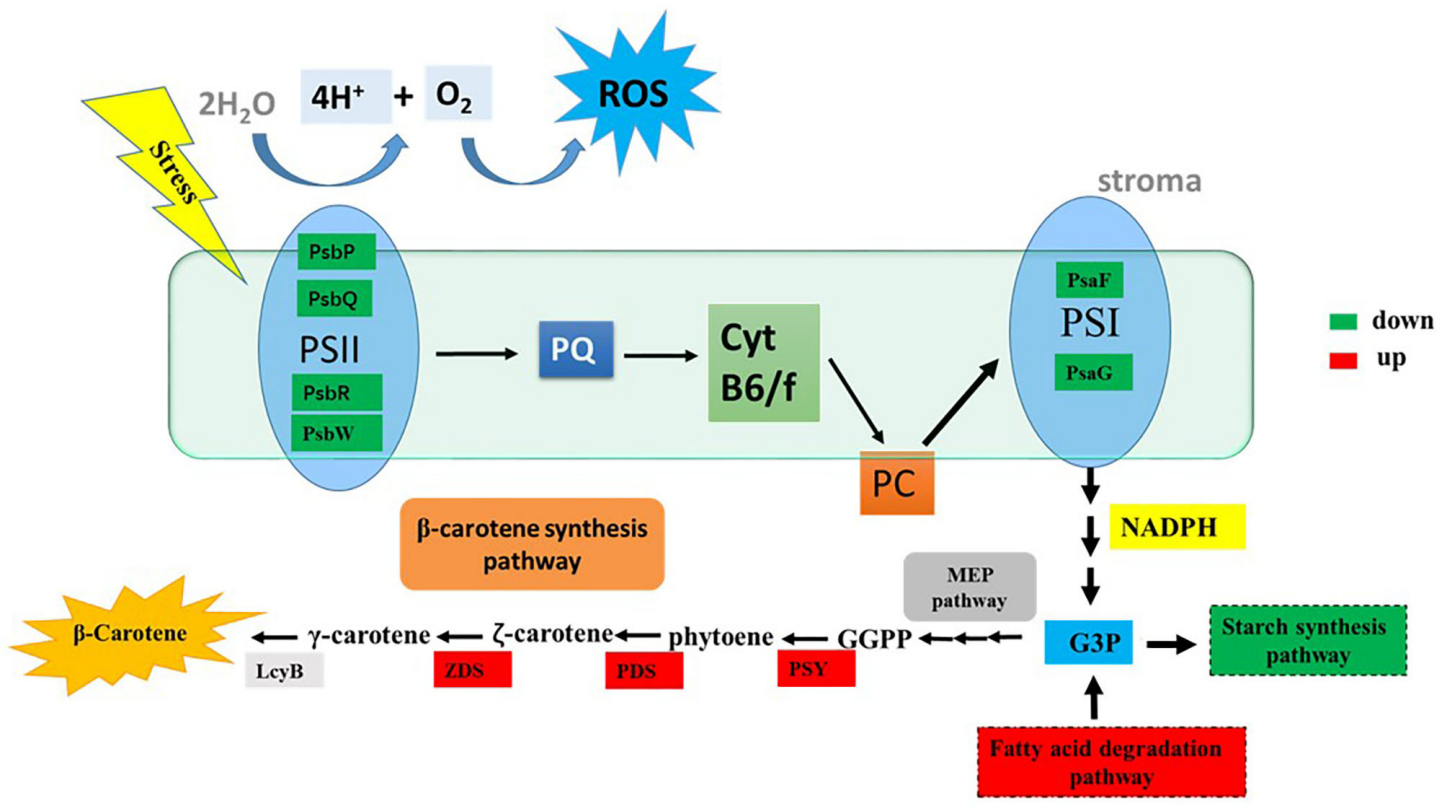
The tentative model of the correlationship between ROS and β-carotene accumulation. G3P, glyceraldehyde-3-phosphate; GGPP, geranylgeranyl diphosphate; LcyB, lycopene β-cyclase; PDS, phytoene desaturase; PSY, phytoene synthase; ZDS, z-Carotene desaturase; PQ, plastoquinone; PC, plastocyanin; ROS, reactive oxygen species; PsbP, photosystem II oxygen-evolving enhancer protein; PsbQ, photosystem II oxygen-evolving enhancer protein; PsbR, photosystem II 10 kDa protein; PsbW, photosystem II reaction center W protein; PsaF, photosystem I reaction center subunit III; PsaG, photosystem I reaction center subunit V. Significantly upregulated and downregulated genes are shown as red and green rectangles.

In this study, the potential relationship between ROS and β-carotene biosynthesis is proposed in *D. salina* under stress conditions (Figure 6). Our results suggested that the key genes involved in photosynthesis pathways were downregulated in the cells when H_2_O_2_ was supplemented (Supplementary Figure 1), which indicated that the photosynthetic activity was reduced. The excessive electrons from the photosynthesis system could lead to ROS generation and overaccumulation (Shaish et al., 1993), which will further result in decreased generation of NADPH and ATP. Therefore, NADPH and ATP might not be sufficient for the synthesis of glyceraldehyde-3-phosphate (G3P, which is the precursor and carbon skeleton for β-carotene biosynthesis) through the Calvin cycle. The downregulated genes for the synthesis of starch and upregulated genes for the fatty acid degradation pathway under H_2_O_2_ supplementation in this study indicate that more cellular energy and precursors might be recycled or shuttled for β-carotene biosynthesis. Overaccumulated ROS are proposed to trigger the high expressions of key genes (e.g., *PSY, PDS, ZDS*, and *LcyB*) that are involved in β-carotene synthesis (Figure 6) and thus lead to β-carotene overaccumulation in *D. salina* under stress conditions. Therefore, it is likely that ROS can also serve as mediators or second messengers and play important roles in the regulation of intracellular energy metabolism in response to environmental stress in microalgae *D. salina*. In this work, we found that β-carotene overaccumulation was induced by ROS through fine-tuning the genes involved in β-carotene biosynthesis. Based on these results, the relationship between ROS and β-carotene accumulation in *D. salina* under stress conditions was proposed. This study provided a better understanding of the regulatory mechanism involved in β-carotene accumulation in *D. salina*, which will be useful for overaccumulation of carotenoids and other valuable compounds in other microalgae.

## Conclusions

In this study, a high positive Spearman correlation coefficient was observed between the cellular ROS level and β-carotene content in *D. salina* under stress conditions. Moreover, our transcriptome data indicate that most of the DEGs involved in biosynthesis of β-carotene were significantly upregulated, and those of which involved in photosynthesis activity were downregulated when the cells cultivated with H_2_O_2_ supplementation. Overall, our results indicated that ROS induce β-carotene biosynthesis caused by changes of photosynthesis efficiency and energy metabolism in *D. salina* under stress conditions.

## Data Availability Statement

The raw data supporting the conclusions of this article will be made available by the authors, without undue reservation.

## Author Contributions

YX, FK, and ZC conceived and designed the experiments. YX performed all the experiments. YX and FK analyzed the data and drafted the manuscript. All authors contributed to the article and approved the submitted version.

## Conflict of Interest

The authors declare that the research was conducted in the absence of any commercial or financial relationships that could be construed as a potential conflict of interest.
