# Supplementary material for: ROS Induce β-Carotene Biosynthesis Caused by Changes of Photosynthesis Efficiency and Energy Metabolism in Dunaliella salina Under Stress Conditions
Source: Front Bioeng Biotechnol. 2021 Jan 15;8:613768. doi: 10.3389/fbioe.2020.613768 (PMC7844308; doi:10.3389/fbioe.2020.613768)
Supplement: Supplementary file 1 [file Table_1.DOCX]

Supplementary Material

**ROS induce β-carotene biosynthesis caused by changes of photosynthesis efficiency and energy metabolism in *Dunaliella salina* under stress conditions**

Yimei Xi, Fantao Kong^*^, Zhanyou Chi

School of Bioengineering, Dalian University of Technology, Dalian 116024, China

# Supplementary Figures


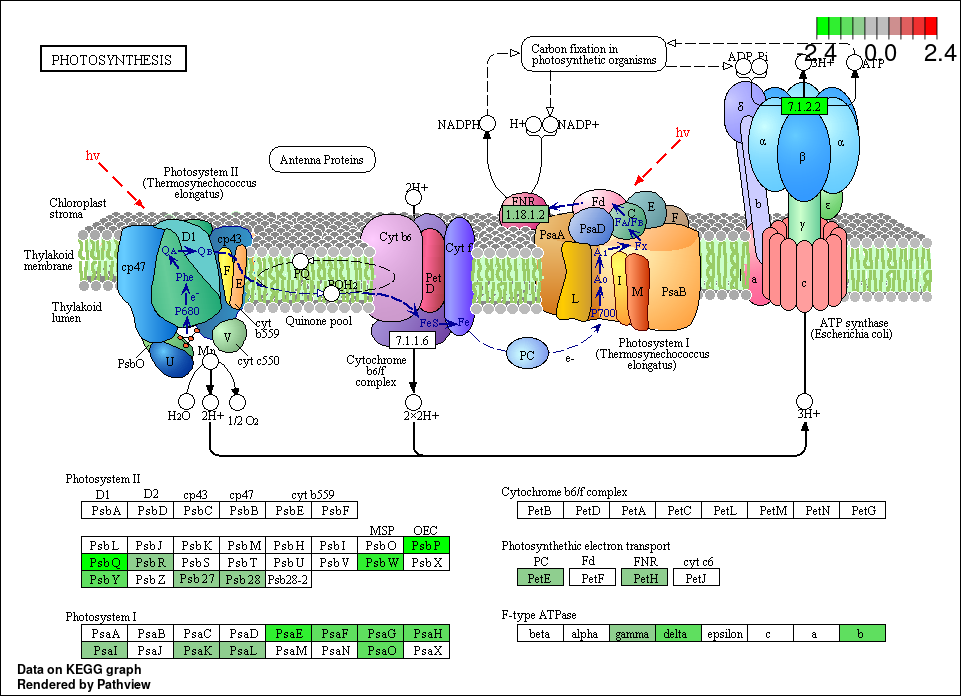


**Supplementary Figure 1.** The representative KEGG-annotated DEGs that were involved in photosynthesis pathways when the *D. salina* cells cultivated with H_2_O_2_ supplementation (2.0 mM) compared with control (0 mM H_2_O_2_). The green color of boxes indicated the down-regulated genes.


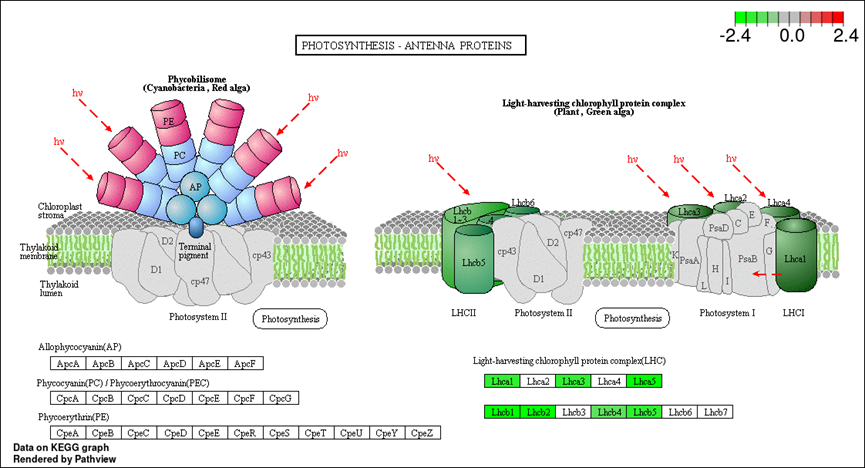


**Supplementary Figure 2.** The representative KEGG-annotated DEGs that were involved in antenna proteins synthesis when the *D. salina* cells cultivated with H_2_O_2_ supplementation (2.0 mM) compared with control (0 mM H_2_O_2_). The green color of boxes indicated the down-regulated genes.


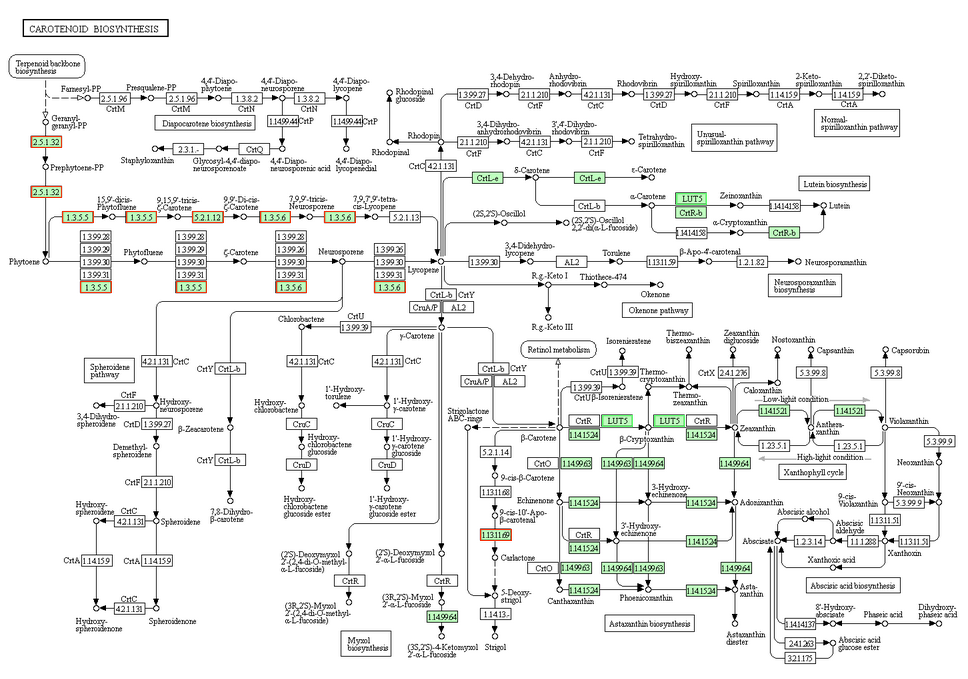


**Supplementary Figure 3.** The representative KEGG-annotated DEGs that were involved in carotenoid biosynthesis pathway when the *D. salina* cells cultivated with H_2_O_2_ supplementation (2.0 mM) compared with control (0 mM H_2_O_2_). The green-colored boxes indicated the down-regulated genes. The green boxes with red-colored frame indicated the up-regulated genes.

##

## Supplementary Tables

## Supplementary Table 1. The representative genes that are down-regulated in starch and sucrose metabolism pathways when the *D. salina* cells cultivated with H_2_O_2_ supplementation (2.0 mM) compared with control (0 mM H_2_O_2_).

| Gene | Description |
| --- | --- |
| ISA | glycosyl transferase[EC:3.2.1.68] |
| GPM | phosphoglucomutase[EC:5.4.2.2] |
| DPE | 4-alpha-glucanotransferase[EC:2.4.1.25] |
| AMYB | beta-amylase[EC:3.2.1.2] |
| AGL | alpha glucosidase[EC:3.2.1.20] |
| SBE | 1,4-alpha-glucan branching enzyme [EC:2.4.1.18] |
| SSS | starch synthase [EC:2.4.1.21] |
| STA | glucose-1-phosphate adenylyltransferase[EC:2.7.7.27] |
| HXK1 | hexokinase[EC:2.7.1.1] |

**Supplementary Table 2.** The representative genes that are down-regulated in amino acids biosynthesis pathways when the *D. salina* cells cultivated with H_2_O_2_ supplementation (2.0 mM) compared with control (0 mM H_2_O_2_)*.*

| Gene | Description |
| --- | --- |
| PFK | 6-phosphofructokinase 1 [EC:2.7.1.11] |
| FBA | fructose-bisphosphate aldolase, class I [EC:4.1.2.13] |
| TPIC | triosephosphate isomerase (TIM) [EC:5.3.1.1] |
| FBA | fructose-bisphosphate aldolase, class I [EC:4.1.2.13] |
| GAP | glyceraldehyde 3-phosphate dehydrogenase [EC:1.2.1.12] |
| PGH | enolase [EC:4.2.1.11] |
| PYK | pyruvate kinase [EC:2.7.1.40] |
| RPE | ribulose-phosphate 3-epimerase [EC:5.1.3.1] |
| RPPK | ribose-phosphate pyrophosphokinase [EC:2.7.6.1] |
| HIS | imidazole glycerol-phosphate synthase [EC:4.3.2.10] |
| HDH | histidinol dehydrogenase [EC:1.1.1.23] |
| TRK | transketolase [EC:2.2.1.1] |
| FSA | transaldolase [EC:2.2.1.2] |
| SHKA | 3-deoxy-7-phosphoheptulonate synthase [EC:2.5.1.54] |
| DHQS | 3-dehydroquinate synthase [EC:4.2.3.4] |
| SHKH | chorismate synthase [EC:4.2.3.5] |
| CHM | chorismate mutase [EC:5.4.99.5] |
| TSA | tryptophan synthase alpha chain [EC:4.2.1.20] |
| AGD | arogenate dehydrogenase (NADP+), plant [EC:1.3.1.78] |
| PRD | arogenate/prephenate dehydratase [EC:4.2.1.91 4.2.1.51] |
| PSP | phosphoserine phosphatase [EC:3.1.3.3] |
| SAT | serine O-acetyltransferase [EC:2.3.1.30] |
| OASTL | cysteine synthase [EC:2.5.1.47] |
| DPD | diaminopimelate decarboxylase [EC:4.1.1.20] |
| ARG | argininosuccinate lyase [EC:4.3.2.1] |
| DPA | LL-diaminopimelate aminotransferase [EC:2.6.1.83] |
| OTC | ornithine carbamoyltransferase [EC:2.1.3.3] |
| GLN | glutamine synthetase [EC:6.3.1.2] |

**Supplementary Table 3.** The representative genes that are down-regulated in fatty acid biosynthesis pathway when the *D. salina* cells cultivated with H_2_O_2_ supplementation (2.0 mM) compared with control (0 mM H_2_O_2_)*.*

| Gene | Description |
| --- | --- |
| FabF | 3-oxoacyl-[acyl-carrier-protein] synthase II [EC:2.3.1.179] |
| FabD | [acyl-carrier-protein] S-malonyltransferase [EC:2.3.1.39] |
| FabG | [3-oxoacyl-[acyl-carrier protein] reductase [EC:1.1.1.100]](https://www.kegg.jp/dbget-bin/www_bget?ec:1.1.1.100) |
| FabZ | [3-hydroxyacyl-[acyl-carrier-protein] dehydratase [EC:4.2.1.59]](https://www.kegg.jp/dbget-bin/www_bget?ec:4.2.1.59) |
| FabI | enoyl-[acyl-carrier protein] reductase I [EC:1.3.1.9 1.3.1.10] |

**Supplementary Table 4.** The representative genes that are up-regulated in fatty acids degradation pathway when the *D. salina* cells cultivated with H_2_O_2_ supplementation (2.0 mM) compared with control (0 mM H_2_O_2_)*.*

| Gene | Description |
| --- | --- |
| FDH1 | S-(hydroxymethyl)glutathione dehydrogenase [EC:1.1.1.284 1.1.1.1] |
| ATO1 | acetyl-CoA acyltransferase 1 [EC:2.3.1.16] |
| ACAT | acetyl-CoA C-acetyltransferase [EC:2.3.1.9] |
| ACSL | long-chain acyl-CoA synthetase [EC:6.2.1.3] |
